# Analyses with double knockouts of the *Bmpr1a* and *Bmpr1b* genes demonstrate that BMP signaling is involved in the formation of precerebellar mossy fiber nuclei derived from the rhombic lip

**DOI:** 10.1371/journal.pone.0226602

**Published:** 2019-12-23

**Authors:** Lihua Qin, Kyung J. Ahn, Lara Wine Lee, Charles de Charleroy, E. Bryan Crenshaw

**Affiliations:** 1 Division of Pediatric Otolaryngology, Mammalian Neurogenetics Group, Center for Childhood Communication, The Children's Hospital of Philadelphia, Philadelphia, Pennsylvania, United States of America; 2 Department of Anatomy and Histoembryology, School of Basic Medical Sciences, Peking University Health Science Center, Beijing, China; 3 Neuroscience Graduate Group, University of Pennsylvania School of Medicine, Philadelphia, Pennsylvania, United States of America; 4 Department of Otorhinolaryngology, Head and Neck Surgery, The Children's Hospital of Philadelphia, Philadelphia, Pennsylvania, United States of America; Tokyo Medical and Dental University, JAPAN

## Abstract

Bone morphogenetic proteins (BMPs) have been hypothesized to specify distinct dorsal neural fates. During neural development, BMPs are expressed in the roof plate and adjacent neuroepithelium. Because several hindbrain nuclei that form the proprioceptive/vestibular/auditory sensory network originate from the rhombic lip, near the roof plate, BMP signaling may regulate the development of these nuclei. To test this hypothesis genetically, we have examined the development of the hindbrain in BMP type I receptor knockout mice. Our results demonstrate that BMP signaling is involved in the formation of precerebellar mossy fiber nuclei, which give rise to cerebellar mossy fibers, but is not required for the development of the inferior olivary nucleus, which gives rise to cerebellar climbing fibers.

## Introduction

The embryonic rhombic lip is a specialized germinative epithelium that arises at the interface between the neural tube and the roof plate of the fourth ventricle. Neuronal precursors generated in the rhombic lip undertake long distance migration to widely dispersed destinations, giving rise to neural cell types in the vestibular/auditory/cerebellar systems [1–3]. Classically, the rhombic lip has been divided into rostral (anterior/upper) and caudal (posterior/lower) parts. Neuronal cell types of the cerebellum and rostral hindbrain originate in the rostral rhombic lip [4–7]. In addition to the cochlear and vestibular nuclei, neurons originating in the caudal rhombic lip form the precerebellar mossy fiber nuclei [3, 8–13]. There are five precerebellar mossy fiber nuclei: the pontine gray (PN) and reticulotegmental nuclei (RtTg), located in the pons; the inferior olivary nucleus (ION), the external cuneate (ECu) and lateral reticular nuclei (LRt), located in the medulla [8–11]. The pontine gray and reticulotegmental nuclei are derived from rhombomeres 6–8, and the other precerebellar mossy fiber nuclei are derived from rhombomeres 7–8 [3]. Axons from these nuclei form the two major inputs into the cerebellum, namely, the mossy and climbing fibers.

The rhombic lip has a dorsal-ventral graded expression of *Wnt1* [14, 15]. The pre-cerebellar progenitor neurons originate within the *Wnt1* expressing caudal rhombic lip and are spatially and molecularly defined. The mossy fiber precerebellar neurons which populate the PN, RtTg, ECu, and LRt originate from the dorsal domain of the caudal rhombic lip specified by high expression of *Wnt1* and the expression of *Atoh1*, a basic helix-loop-helix transcription factor [3, 6, 8–11, 14–18]. Mutations in the *Atoh1* gene result in the loss of precerebellar mossy fiber nuclei from which the mossy fiber input to the cerebellum originate [19]. Climbing fiber neurons which contribute to the ION are derived from a more ventral domain of the caudal rhombic lip which expresses a low level of *Wnt1* and also expresses *Ptf1a* (pancreatic transcription factor 1a) [2,3, 9, 18, 20–25]. The *Pft1a* null mutant lacks the ION, which form climbing fiber neurons, but not the other precerebellar mossy fiber nuclei [23]. These data demonstrate that the initial dorsal-ventral patterning of the rhombic lip plays a crucial role in specifying cell types in the precerebellar system. For this reason, we have characterized the development of the precerebellar system in mouse mutants that abrogate BMP signaling–a signaling pathway that plays crucial roles in dorsal-ventral patterning in the neural tube [25, 26].

BMP gene family members, which belong to the transforming growth factor-β (TGF-β) superfamily, regulate a number of cell processes during development, including differentiation, cell growth, apoptosis, cell-fate determination, and morphogenesis [27,28]. BMPs are expressed in the roof plate and in the adjacent dorsal neural tube [25, 29, 30]. Therefore, they have been hypothesized to play a role in regulating the development of the rhombic lip and its derivatives, including the precerebellar mossy fiber nuclei. BMPs have been shown to signal via hetero-oligomeric complexes of transmembrane serine/threonine kinase type I and type II receptors [26, 31–33]. The BMP type I receptors *Bmpr1a* and *Bmpr1b* directly phosphorylate the Smad proteins [34, 35]. The genes for these BMP type I receptors are expressed widely throughout most of neural tube development during every stage of development [36,37]. *Bmpr1a* is expressed extensively throughout development, whereas *Bmpr1b* is expressed in a more limited, but still widespread distribution [36,37]. To genetically characterize the functions of BMP signaling during mouse neural tube development, we have generated a strain of mutant mice containing a double knockout of the genes encoding the BMP receptor-IA and BMP receptor-IB subunits (*Bmpr1a;Bmpr1b* mutant mice, which we refer to as *Bmpr* double knockout mice). Our results demonstrate that BMP signaling through *Wnt1* is involved in the development of precerebellar mossy fiber nuclei that contribute to the mossy fiber inputs into the cerebellum, but not the inferior olivary nucleus, the precerebellar nucleus that contributes climbing fiber inputs.

## Methods

### Mouse strains

The *Bmpr1a* conditional knockout mice were generated as described previously [38, 39]. Briefly, a floxed allele of the *Bmpr1a* gene was conditionally inactivated with the Bcre-32 pedigree. In this pedigree, expression of the Cre recombinase gene was driven by the neural tube enhancer transcriptional regulatory elements of the POU-domain gene, *Brn4/Pou3f4*, Tg(Pou3f4-cre)32Cren, which we will refer to as Bcre-32 [40]. *Bmpr1b* null mutants, a classical knockout, were a kind gift from Karen Lyons [41]. BMP signaling mutants containing double knockouts of the *Bmpr1a* and *Bmpr1b* genes were generated as described previously [40]. Briefly, a floxed allele of the *Bmpr1a* gene is conditionally inactivated in the neural tube by the Bcre-32 transgene in a background containing the *Bmpr1b* homozygous knockout.

To examine the expression domain induced by the Bcre-32 transgenic, the transgenic line was crossed to the Gt(ROSA)26Sor reporter strain, which we will refer to as the ROSA reporter strain. Gt(ROSA)26Sor was developed as a gene-trap allele whose expression can be activated in all cells in the mouse in which the floxed allele is activated [42].

### Ethics statement

The experimental design used in these studies was approved by the Institutional Animal Care and Use Committee (IACUC) at The Children’s Hospital of Philadelphia (IACUC protocol #588).

### Tissue preparation

Embryos were staged by designating the morning of the vaginal plug as 0.5 dpc (days post-coitus). Whole brains were dissected and washed in cold PBS, followed by fixation in 4% paraformaldehyde at 4°C overnight. Embryos were treated with 30% sucrose in PBS at 4°C overnight, embedded in OCT compound (Tissue Tek), and cryosectioned at a thickness of 25 μm for in situ hybridization and immunohistochemistry analyses. Newborn mice were anesthetized, transcardiacally perfused with 0.1M PBS pH 7.3, followed by perfusion with 4% paraformaldehyde in PBS. Subsequently, the brains were dissected and fixed in 4% paraformaldehyde at 4°C overnight. Yolk sacs or tails were collected prior to fixation for DNA extraction and genotyping by PCR analysis. Primers for genotyping the alleles of *Bmpr1a*, *Bmpr1b*, and Bcre-32 were described previously [40]. For paraffin sections, fixed brains were dehydrated and processed through paraffin and embedded via standard procedures. Tissue sections (7 μm) were used for histological analysis.

### In situ hybridization

Digoxygenin-labeled RNA probes were synthesized as run-off transcripts from linearized plasmid templates using RNA polymerases (T3, T7, and Sp6) according to the manufacturer’s protocol (Roche). The following antisense probes were used: *Atoh1*(gift of J. Johnson), *Barhl1* (IMAGE clone 335997 linearized with StuI), *Ngn2* (gift of D. Anderson), *Pax6* (gift of M. Goulding), *Ptf1a* (IMAGE clone 5942372 linearized with PstI) and *Rph3a* (IMAGE clone 5698459 linearized with XbaI). In situ hybridization was accomplished as previously described [40, 43]. Three to five animals from each age and genotype were examined.

### Immunohistochemistry and histology

Single-label immunohistochemistry was performed by incubating overnight at 4°C with a rabbit anti-Zic 1/2 antibody (1:400, a gift from Rosalind Segal, Dana Farber Cancer Institute). Double-label immunohistochemistry was performed using a mouse monoclonal anti-TAG-1/4D7(1:50, DSHB) with a rabbit anti-Zic 1/2 (1:400) by incubating overnight in PBS/2% goat serum/0.1% Triton X-100. Fluorescence-conjugated secondary antibodies consisting of goat anti-rabbit IgG rhodamine and goat anti-mouse IgM FITC (Jackson Immunoresearch) were used. Following a final wash with PBS, nuclei were visualized by staining with DAPI (4',6-diamidino-2-phenylindole, Sigma).

Phospho-Smad immunohistochemical analyses were accomplished using previously published methods [38, 40]. Lhx1/5, Pax2 double-immunolabel immunohistochemistry was performed as previously described [40]. For these double-label analyses, sections from the hindbrain region caudal to the otic vesicle–corresponding to a region of the hindbrain derived from rhombomere 6—was analyzed, and the pattern of Lhx1/5 and Pax2 expression was used to confirm the identity of the sections [44]. Histological analyses of neonatal mice brains were performed on paraffin sections using Nissl staining and staining with hematoxylin and eosin (H&E).

Staining for lacZ expression using X-gal (5-bromo-4-chloro-3-indolyl-b-D-galactopyranoside) was accomplished as described previously [45]. Three to five animals from each age and genotype were examined.

Apoptosis and cell proliferation in 10 dpc hindbrain were assessed respectively by terminal deoxynucleotidyl transferase mediated dUTP nick end-labeling (TUNEL) assay (Roche) and anti-phospho-histone H3 (1:200; Upstate Biotechnology) as previously described [46]. Quantitative analyses of TUNEL and phospho-histone H3 were undertaken by counting the percentage of TUNEL positive or phospho-histone H3 positive cells in the area where *Atoh1*is expressed in normal embryos. For *Bmpr* double knockouts, where *Atoh1* is not expressed, the area equivalent to the *Atoh1-*positive cells of a corresponding normal littermate was analyzed. At least 8 normal and mutant littermates were examined.

## Results

### BMP signaling is abrogated in the caudal rhombic lip region in *Bmpr* mutant animals

Conditional knockouts were induced using a transgenic pedigree, Bcre-32, that contains the neural enhancer/promoter from the *Brn4/Pou3f4* gene driving the expression of Cre recombinase [38, 40, 46]. This transgenic strain contains 6 kb of the *Brn4/Pou3f4* promoter region driving the expression of the cre recombinase gene is expressed initially in the neural plate beginning at 8.5 dpc and continues to be expressed in the neural tube throughout embryogenesis. Conditional knockout of the *Bmpr1a* gene alone using the Bcre-32 transgene results in a viable mutant with a limb phenotype [38] and hydrocephalus (S1 Fig); we have not observed any neuroanatomical differences in the hindbrain of this single mutant. The knockout mutation of the *Bmpr1b* is also viable with limb and fertility defects [41], also suggesting that there are no gross neuroanatomical malformations in these mutants either.

**Fig 1:**
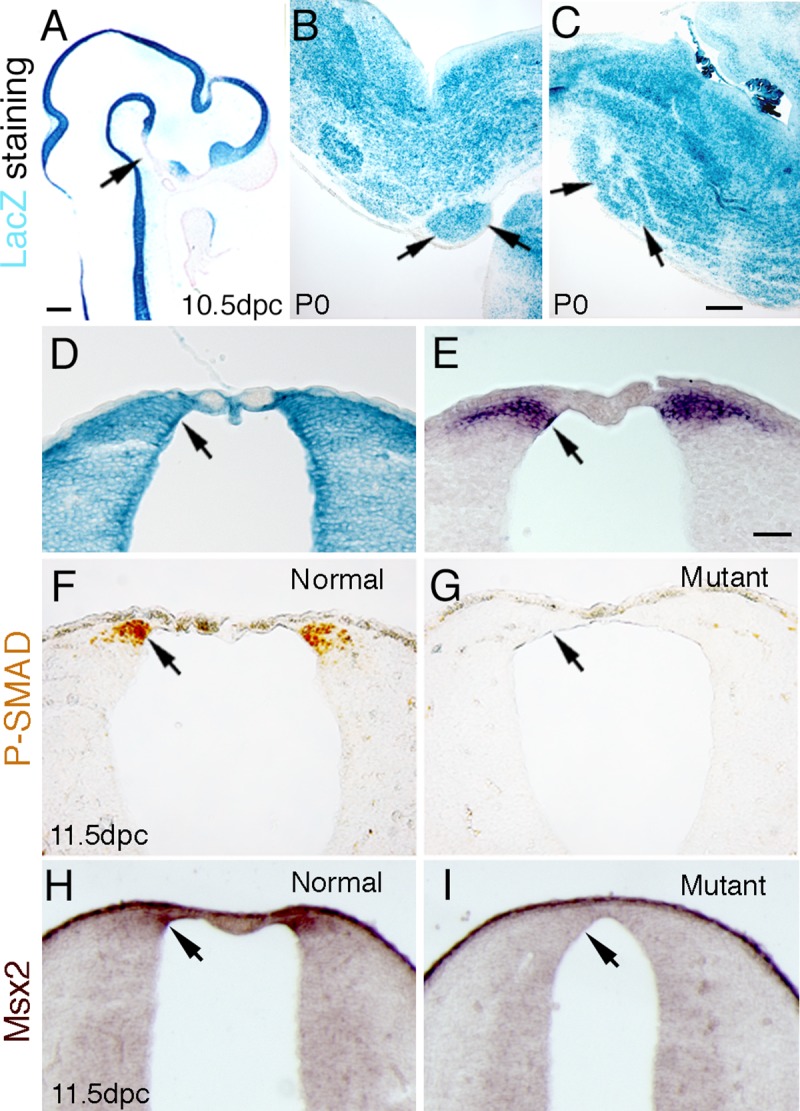
Cre-mediated recombination in the hindbrain activates lacZ expression from the ROSA reporter and abrogates Phospho-Smad immunostaining in conditional *Bmpr* double knockout mutants. The Bcre-32 transgenic line induces the expression of lacZ throughout most of the neural tube at 10.5 dpc, except the ventral diencephalon (A, arrow). LacZ expression was detected in the pontine gray nucleus postnatally (B, arrow), and in the inferior olivary nuclei at P0 (C, arrow). In Panel D, a coronal section of 10.5 dpc caudal hindbrain demonstrates that lacZ expression is observed in the region of the dorsal hindbrain that expresses Atoh1, as shown by in situ hybridization in Panel E (compare arrow in E to arrow in D). Panel F demonstrates that phospho-Smad immunolabelled cells were found in the caudal rhombic lip at 11.5 dpc in the normal animals (arrow). However, no phospho-Smad-positive cells were detected in the same region and the same stage in the *Bmpr* double knockout animals (G, arrow). Panel H demonstrates the expression pattern of Msx2, whose expression is directly downstream of BMP signaling, in a normal 11.5 dpc embryo. In the mutant (Panel I), the expression of Msx2 was not detected in the neural tube. Scale bar: A, 250 μm; C (for B,C), 500 μm; E (for D-I), 50 μm.

To determine the spatial and temporal expression of the Bcre-32 gene in the precerebellar mossy fiber nuclei, we intercrossed the Bcre-32 pedigree with the ROSA reporter strain, which activates the expression of the lacZ gene upon Cre-mediated recombination [42]. At 10.5 dpc, Bcre-32-mediated expression of lacZ is detected throughout most of the neural tube including the caudal rhombic lip (Fig 1A and 1D). Coronal sections of 10.5 dpc embryos demonstrate that Cre-mediated expression of lacZ is in the caudal rhombic lip of the hindbrain (Fig 1D, arrow) in regions that encompass the expression domain of *Atoh1* (compare arrows in Fig 1D and 1E). Postnatally, the hindbrain, including the pontine gray nuclei and inferior olivary nucleus, express the lacZ reporter (Fig 1B and 1C, arrow). Previously, we have demonstrated by Southern blot analysis that the Bcre-32 transgene induces Cre-mediated deletion of the *Bmpr1a* gene in >95% of the cells in the hindbrain and spinal cord [38]. These data demonstrate that the Bcre-32 transgene efficiently induces gene inactivation of *Bmpr1a* in the region of the neural tube that will give rise to the hindbrain.

To directly assess the loss of BMP signaling, the expression of phosphorylated Smad (phospho-Smad) was examined. At 11.5 dpc, phospho-Smad immunopositive cells were detected in the caudal rhombic lip in normal embryos (Fig 1F). However, no labeled cells were detected in the caudal rhombic lip of *Bmpr1a;Bmpr1b* knockout mutants (*Bmpr* double knockout mice) at the same stage (Fig 1G). An additional confirmation of the loss of BMP signaling is demonstrated by the loss of expression of the *Msx2* gene (Fig 1H and 1I), whose expression is directly downstream of BMP signaling. These data demonstrate that loss of both type I receptors abrogates BMP signaling in the caudal rhombic lip.

### The expression of genes involved in the specification of rhombic lip progenitors are not detected in *Bmpr* double knockout animals

**Fig 2:**
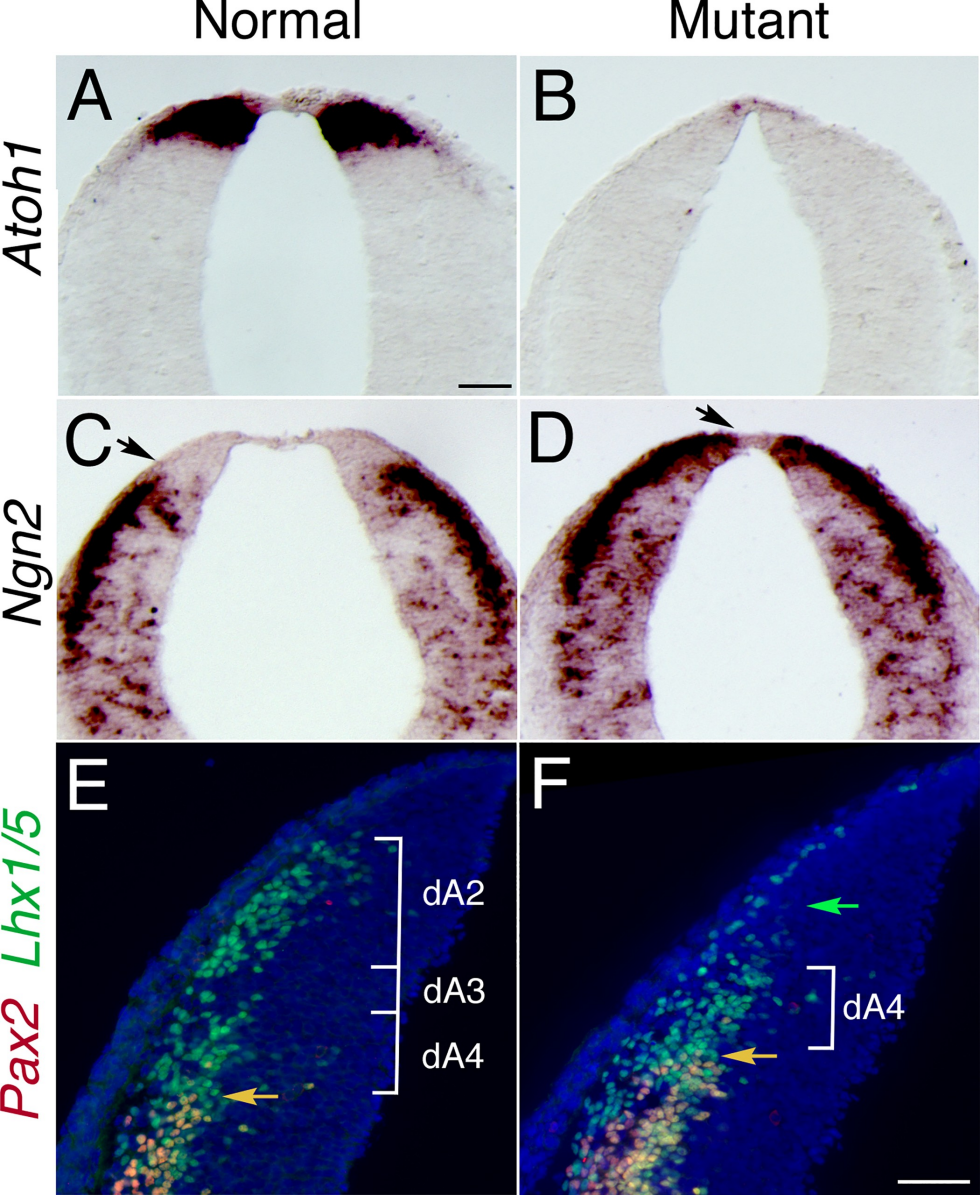
Expression of specific markers of rhombic lip demonstrates that the most dorsal precursors were lost and that ventral markers were shifted dorsally in the caudal rhombic lip in *Bmpr* double knockout animals. (A) *Atoh1* expression in the caudal rhombic lip in normal animal at 10.5 dpc. *Atoh1* expression was essentially lost in *Bmpr* double knockouts at the same stage (B). (C) *Ngn2* expression in normal animals. (D) *Ngn2* expression was shifted dorsally in the *Bmpr* double knockout animals (arrow marks the dorsal extent of expression in C, D). (E, F) Expression of Lhx1/5 and Pax2 demonstrate a vast reduction in the dorsal-most Lhx1/5-labled population (referred to as dA2 from reference 70; green arrow in F), and dorsal shift in class B neurons (orange arrow), which double-label for Lhx1/5 and Pax2, in the *Bmpr* mutant. Scale bar: A (for A-D), 50 μm; F (for E-F), 25 μm.

Previous studies have demonstrated the essential role of *Atoh1* in the formation of rhombic lip derivatives [6, 17, 19, 25, 47]. BMP signaling has been shown to regulate the expression of *Atoh1* in the spinal cord [40, 48, 49]. In *Bmpr* double knockout animals, *Atoh1* expression was lost in the caudal rhombic lip at 10.5 dpc (Fig 2A and 2B).

The transcription factor *Ngn2* is important for the maintenance of neuronal precursors and specification of cell fates at a level more ventral than the *Atoh1* expression domain [50]. To determine whether more ventral precursors were affected in the *Bmpr* double mutants, we examined the expression of *Ngn2*. In comparison with normal animals, the expression domain of *Ngn2* at 10.5 dpc in the *Bmpr* double knockout animals was shifted dorsally (Fig 2C and 2D).

To further examine the changes that have occurred in dorsal interneuron cell types in the hindbrain, we have undertaken double-label immunofluorescent analyses using antibodies directed against Pax2 and Lhx1/5. This approach gives a distinctive pattern of labeling that can distinguish changes in interneurons in the region of rhombomere 6 of the hindbrain [44]. In the normal hindbrain, Pax2/Lhx1/5 double label the ventral-most dorsal interneurons (referred to as B class interneurons in ref. 70), whereas Lhx1/5 alone labels dorsal-most interneuron classes (referred to as dA2 and dA4 in Fig 2E). In the mutant, the upper dorsal population is missing (green arrow in Fig 2F).

To determine if the dorsal shift of *Ngn2* was due to the *Atoh1* expressing cells either undergoing apoptosis or ceasing proliferation due to the loss of BMP signaling, we performed TUNEL assays and phospho-histone H3 immunohistochemistry in the dorsal area of the caudal hindbrain where *Atoh1* is expressed. There were few TUNEL-positive cells in the dorsal *Atoh1* expressing area of the caudal rhombic lip in both the mutant and normal at 10 dpc, and no significant difference in normal and mutant animals (Table 1). There was also no difference in cell proliferation in the *Atoh1* expressing domain between normal and mutants as demonstrated through phospho-histone H3 immunohistochemistry (Table 1). These results demonstrate that changes in the specification of the caudal rhombic lip can be detected as early as 10.5 dpc, resulting in a reduction of the pontine and other precerebellar nuclei that generate mossy fiber innervation of the cerebellum in double knockout animals.

**Table 1:** Apoptosis and cell proliferation are not statistically different in the rhombic lip of normal and mutant animals. Normal-mutant pairs (8–10) of littermates from 8 litters were analyzed for apoptosis by TUNEL assay and for cell proliferation by phospho-Histone3 immunohistochemistry as described in the Methods section. Between 76 and 104 sections were examined and the number of positive cells that labeled in the relevant assay in each section were scored. The average number of positive cells per section are shown with the standard deviation (SD). The statistical significance for each grouping (Assay and Genotype) were assessed by Student t-test.

*Assay*	*Genotype*	*Sections Counted*	*Positive Cells*	*Positive/ section*	*SD*	*p-value*
TUNEL	Normal	104	14	0.12	0.15	
TUNEL	Mutant	84	20	0.21	0.36	0.46
Phospho-H3	Normal	76	322	4.88	1.66	
Phospho-H3	Mutant	89	442	4.70	1.57	0.82

**Fig 3:**
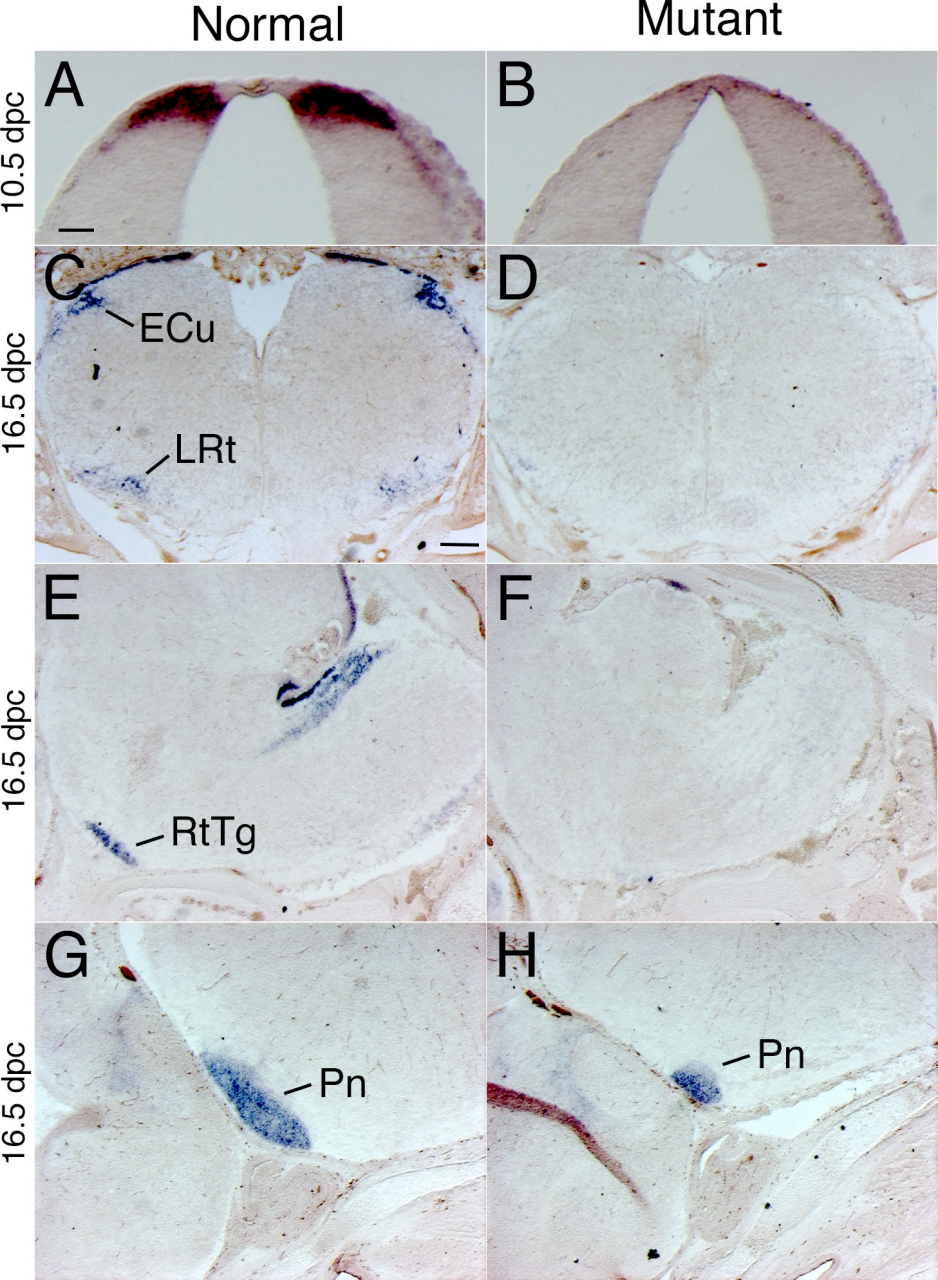
*Barhl1* in situ hybridization demonstrates that the dorsal-most neuroblast population was lost or drastically reduced throughout embryonic development. (A) *Barhl1* is expressed in the dorsal-most neuroblast population of the caudal rhombic lip. *Barhl1* expression is detected in only a few cells in the dorsal hindbrain of *Bmpr* double knockout mutants (B). At 16.5 dpc, *Barhl1*-positive cells were detected in the external cuneate (ECu; Panel C), lateral reticular (LRt; Panel C), reticulotegmental (RtTg; Panel E) and pontine gray nuclei (Pn; Panel G). These cells were largely missing or drastically reduced in the *Bmpr* mutant (D, F, H). All panels in the figure show in situ hybridization, although there is some variability in the precipitate generated by the color reaction chemistry. Scale bar: A (for A, B), 50 μm; C (for C-H), 200 μm.

To further characterize the fate of the rhombic lip precursors, we have examined the expression of a marker gene, *Barhl1*, that is initially expressed in *Atoh1*-expressing rhombic lip precursors and maintains its expression in the postmitotic neuroblasts derived from these precursors [51]. At 10.5 dpc, *Barhl1* is expressed in rhombic lip precursors (Fig 3A) and drastically reduced in *Bmpr* double knockout mutants (Fig 3B). By 16.5 dpc, most of the migratory rhombic lip precursors have populated their mature position in the hindbrain at the external cuneate, lateral reticular, reticulotegmental and pontine gray nuclei (Fig 3C, 3E and 3G). Expression of *Barhl1* is drastically reduced in the hindbrain of *Bmpr* double knockout mutants (Fig 3D, 3F and 3H).

These data are demonstrate that precerebellar precursors that give rise to mossy fiber afferents are greatly reduced in the *Bmpr* double knockout mutants.

### Abnormal development of precerebellar nuclei that generate mossy fibers in *Bmpr* double knockout animals

**Fig 4:**
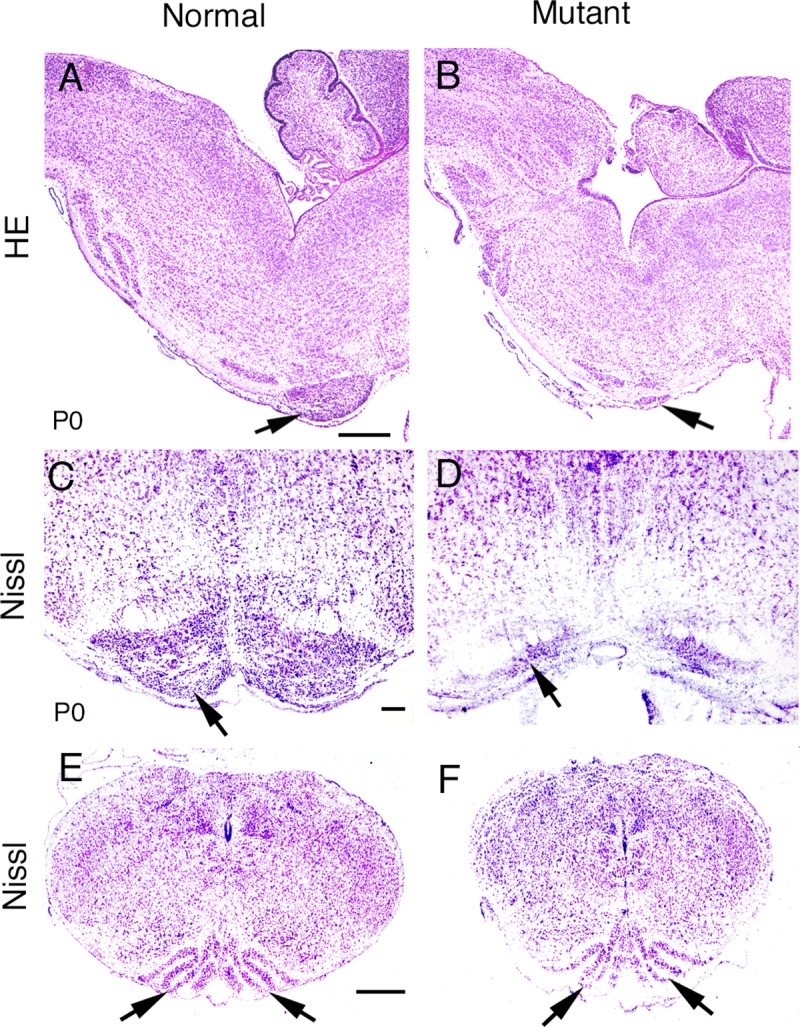
Morphological changes in the hindbrains of *Bmpr* knockout mutants. When BMP signaling was eliminated, most of the pontine gray nucleus was lost (A-D), while the morphology of the inferior olivary nucleus appeared unaffected (E, F). (A, B) H&E staining shows that the pontine gray nucleus was dramatically decreased in the *Bmpr* double knockout mutant (B, arrow) compared to normal (A, arrow). (C, D) Nissl staining of coronal sections shows similar results. Arrows indicate the pontine gray nuclei. (E, F) Arrows indicate the inferior olivary nuclei in both *Bmpr* double knockout (F) and normal (E) animals. Scale bar: A (for A, B), 500 μm; C (for C,D), 100 μm; E (for E,F), 400 μm.

BMP signaling has been shown to be critical for patterning and development of the embryonic neural tube [25, 29, 47]. Because of the importance of BMP signaling in nervous system development, we examined the formation of the hindbrain in *Bmpr* double knockout mice. Histological staining demonstrated severe reductions in the size of the pontine gray nuclei in *Bmpr* double knockout neonates at P0 (Fig 4A and 4B, arrows). When Nissl staining was used to examine cellular detail, the dramatic reduction of neurons of the pontine gray nuclei was evident in the *Bmpr* double knockout animals as compared to normal controls at P0 (Fig 4C and 4D). Other hindbrain nuclei that contribute to mossy fiber innervation of the cerebellum are also drastically reduced or missing in the mutants, including the reticulotegmental, external cuneate and lateral reticular nuclei (Fig 3). A further examination of hindbrain structures revealed a grossly normal inferior olivary nucleus, as shown by Nissl staining, in normal and mutant mice at P0 (Fig 4E and 4F).

*Zic* genes, which encode zinc finger proteins, function in embryonic pattern formation, in the early stages of central nervous system neurogenesis, and in cerebellar maturation [52,53]. *Zic1* and *Zic2* are both strongly expressed in differentiated cells of the hindbrain [53]. Therefore, we examined the expression of *Zic* genes in normal and mutant mice. Our data show that *Zic*-positive cells are found in the pontine nuclei (S2A Fig). In double mutant animals, reduction of *Zic*-positive cells was observed at P0 (S2B Fig).

Another transcription factor, *Pax6*, is expressed in the pontine gray nuclei (for review, Wingate, 2001). *Pax6* expression was detected in cells of the pontine gray nuclei in normal animals by in situ hybridization (S2C Fig). The number of *Pax6*-labeled cells was greatly decreased in the double knockout animals at P0 (S2D Fig).

These results demonstrate that precerebellar nuclei that generate mossy fiber input to the cerebellum are drastically reduced in the *Bmpr* double knockout animals.

### Characterization of inferior olivary nucleus development in *Bmpr* double knockout mutants

**Fig 5:**
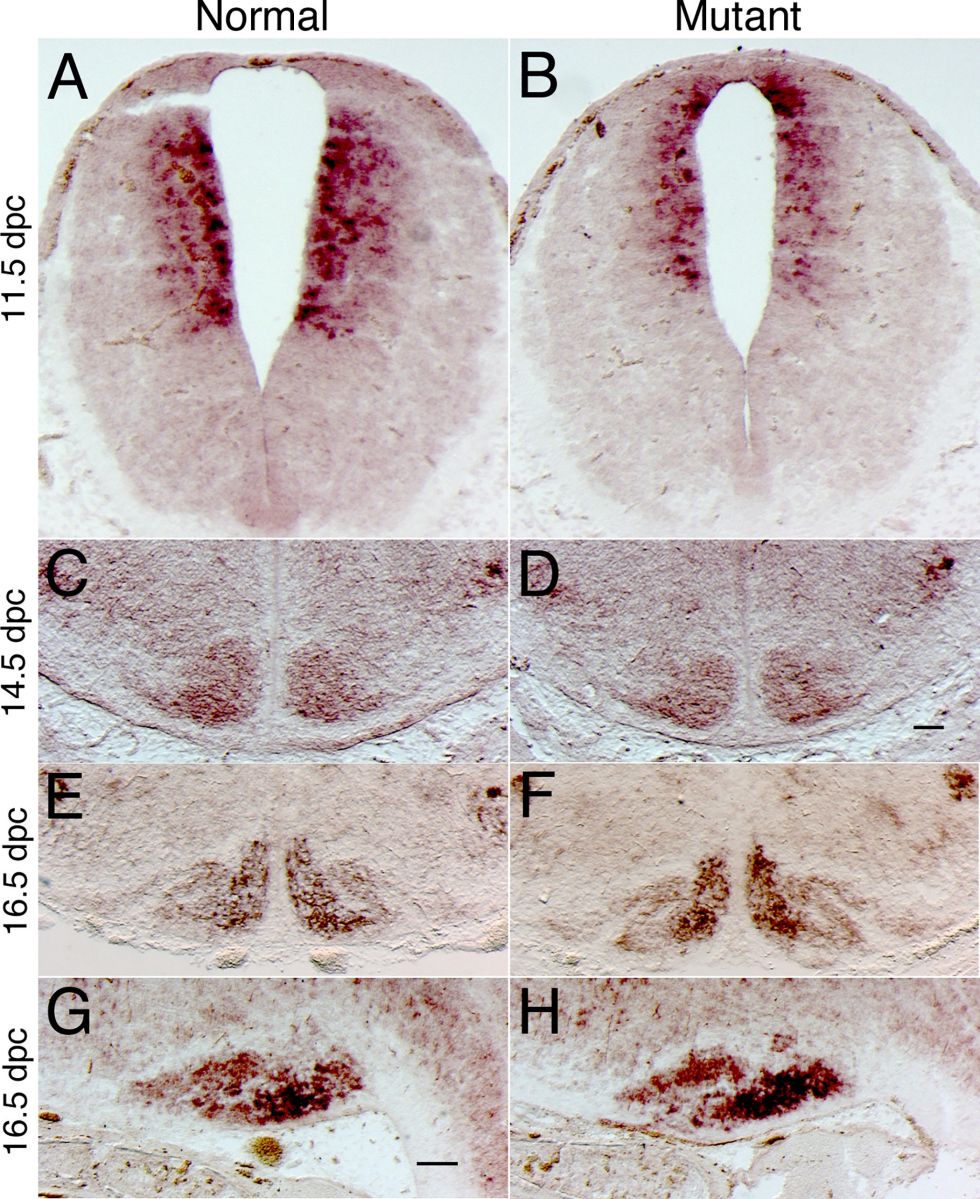
Molecular markers of inferior olive development were initially shifted dorsally, but subsequently, were unaltered. (A) Coronal sections demonstrate that the expression of *Ptf1a* in the ventral alar plate was expanded dorsally in *Bmpr* double knockout mutants (B). Nonetheless, the expression of *Rph3a* was not altered at 14.5 dpc (C, D) or 16.5 dpc (E, F) embryos or in sagittal sections of 16.5 dpc hindbrains (G, H). Scale bar: D (for C, D), 100 μm; G (for E-H), 100 μm.

To examine whether the precerebellar nuclei that give rise to climbing fiber afferents develop properly, we have examined molecular markers of inferior olivary nucleus development [22, 23]. The ION is formed from a region of the embryonic hindbrain that expresses *Ptf1a* [23], which is initially expressed in a dorsolateral domain that lies below the domain expressing *Atoh1* in normal embryos (Fig 5A). In 11.5 dpc *Bmpr* double knockout mutants, the *Ptf1a* expression domain is shifted dorsally, and abuts the roof plate (Fig 5B). However, at 14.5 dpc, another marker of ION development, *Rph3a* [54], is grossly unaltered (Fig 5C and 5D). Furthermore, at 16.5 dpc, the expression domain of *Rph3a* appears unaltered in both coronal (Fig 5E and 5F) and parasagittal sections (Fig 5G and 5H). Therefore, these data demonstrate that, although the embryonic domain that gives rise to the ION is initially shifted dorsally in the *Bmpr* double knockout mutants, this change is transient and does not result in any permanent alteration in the development of the ION.

### The expression of *Wnt1* gene is decreased in *Bmpr* double knockout animals

**Fig 6:**
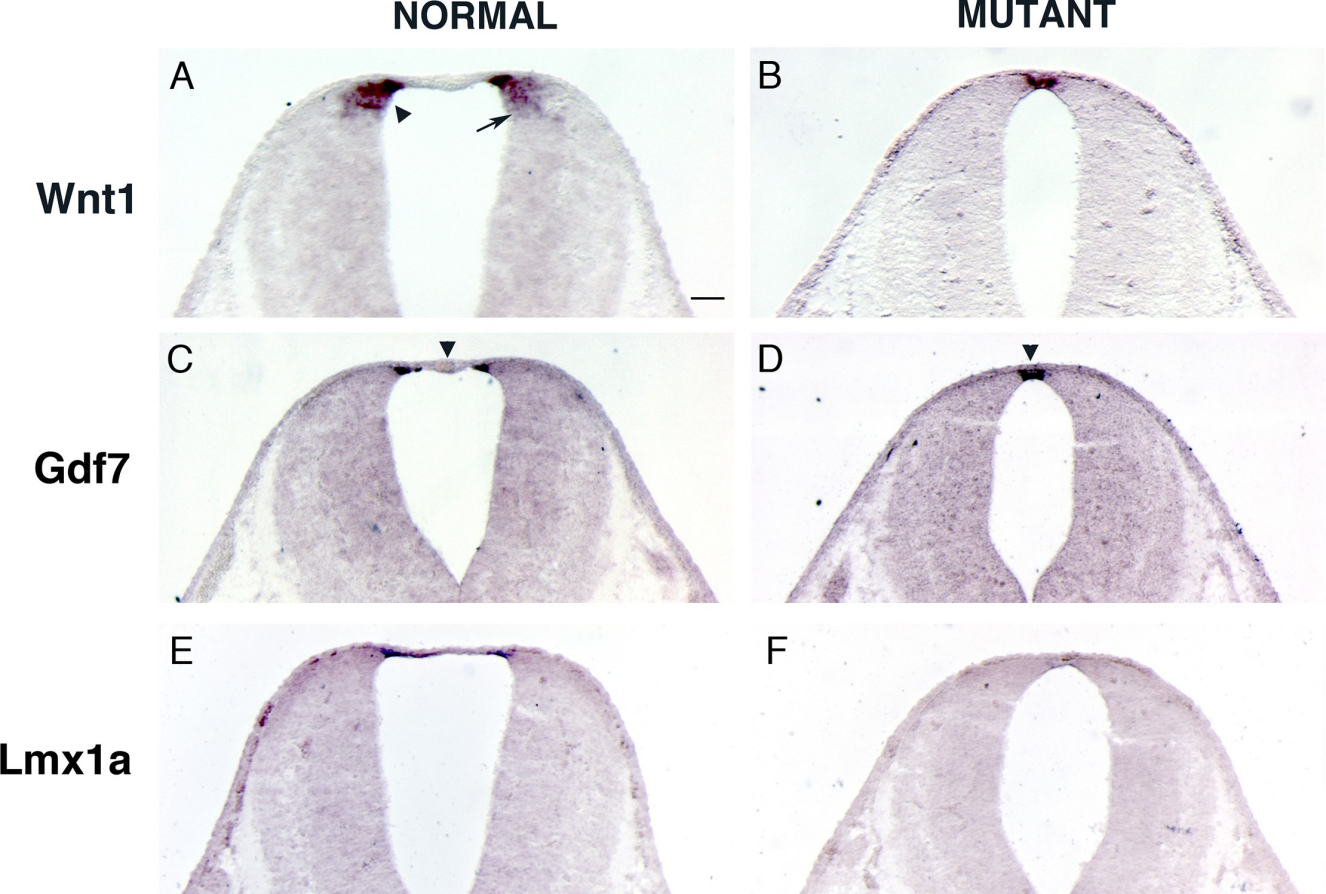
The expression of *Wnt1* gene was decreased in *Bmpr* double knockout mutants. (A) *Wnt1* expression in the caudal rhombic lip in normal animal at 11.5 dpc. The caudal rhombic lip has a dorsal-ventral graded expression of *Wnt1*. The mossy fiber precerebellar neurons originate from the dorsal domain of the caudal rhombic lip specified by high expression of *Wnt1* (arrowhead). Climbing fiber neurons are derived from a more ventral domain of the caudal rhombic lip which expresses a low level of *Wnt1* (arrow). (B) The domain of *Wnt1* expression was reduced in *Bmpr* double knockout animals. (C, D) *Gdf7*, which is expressed in the lateral roof plate, is present in both the normal animals and *Bmpr* double knockout mutants at 11.5 dpc. The non-*Gdf7* expressing medial roof plate was missing in *Bmpr* mutant (compare arrowhead in C and D). (E, F) *Lmx1a*, which is expressed in both the lateral and medial roof plate, was significantly reduced in *Bmpr* mutant at 11.5 dpc. Scale bar: A (for A-F), 50 μm.

The Wnt gene family encodes a group of cysteine-rich secreted glycoproteins involved in a wide range of activities during embryogenesis [55–57]. Members of the Wnt family are expressed in the dorsal neural epithelium and roof plate [58, 59]. Therefore, we examined the effects of BMP signaling abrogation on *Wnt1* gene expression. The roof plate and adjacent dorsal neuroepithelium showed *Wnt1* expression in normal animals at 11.5 dpc (Fig 6A). The domain of *Wnt1* expression was reduced in *Bmpr* double knockout animals (Fig 6B). Thus, our results indicate that BMP signaling is necessary for appropriate expression of *Wnt1* gene in the hindbrain.

The remaining *Wnt1* expression in *Bmpr* double knockout animals seems to be expressed in the roof plate (Fig 6B). Therefore, we examined the expression of *Gdf7*, which is expressed in the lateral roof plate (Fig 6C), and *Lmx1a*, which is expressed in both the lateral and medial roof plate (Fig 6E, [60]). *Gdf7* is present in the *Bmpr* mutant (Fig 6D) and seems to mirror *Wnt1* expression in the mutant (compare Fig 6B to Fig 6D). However, *Lmx1a* is drastically reduced in the mutant (Fig 6F). Also, non-*Gdf7* expressing medial roof plate is reduced in the *Bmpr* double knockout (arrowhead in Fig 6C and 6D).

## Discussion

### BMP signaling through BMP type I receptors regulates the development of the hindbrain

BMPs are multifunctional proteins that specify the fate of different cell types [34]. Several lines of evidence indicate that individual or combinatorial actions of BMPs specify distinct dorsal neural fates [25, 29, 47]. BMP signaling through BMP type I receptors is required for the specification of the dorsal commissural neurons in the spinal cord [40]. Several classes of hindbrain cell types, including the granule cells of the cerebellum and precerebellar mossy fiber nuclei, originate from the rhombic lip [8–11]. The neurons of the pontine gray nuclei appear to be drawn from a large extent of the hindbrain rhombic lip [1, 61, 62]. Studies have shown that the transcription factor *Atoh1* is required for the production of differentiated neurons generated from the rhombic lip [19]. Our results demonstrate that loss of BMP signaling abrogates the formation of *Atoh1*-positive cells in the caudal rhombic lip.

BMPs exert their effects through distinct combinations of types I and II serine/threonine kinase receptors that phosphorylate and thereby activate their nuclear effectors, termed Smads [34, 63]. Thus, we used an immunohistological assay that specifically detects the phosphorylated form of Smad1/5/8 to evaluate BMP signaling. Our data indicated that phospho-Smads were not detected in dorsal neural precursors of *Bmpr* double knockout animals at 11.5 dpc (Fig 1F and 1G; [40]), thus demonstrating that Smad-mediated BMP signaling has been lost. Subsequently, only a vanishingly small number of *Atoh1*-positive cells are detected in the hindbrain of *Bmpr* knockout mutants. In its place, we observe a dorsal shift in the domain of more ventral early markers, such as *Ngn2* (Fig 2D) and *Ptf1a* (Fig 5B).

Our hindbrain analyses were facilitated by following the expression of the *Barhl1* gene, which is initially expressed in the rhombic lip and continues to be expressed in neuroblasts born from rhombic lip precursors (as shown in Fig 3). We detected a drastic down-regulation of *Barhl1* in the *Bmpr* mutant, indicating that *Barhl1* precursors are not specified appropriately. We did not observe ectopic expression of *Barhl1*, arguing against the hypothesis that the cells are specified, but do not migrate properly. Furthermore, developmental delay is unlikely, because we did not observe any *Barhl1* cells in the migratory streams that lead to the pre-cerebellar mutants (Fig 3). Finally, it is unlikely that the cells are specified and then undergo apoptosis, because we do not detect an increase in TUNEL-positive cells in the mutant (Table 1). Nor is it likely that the decreased *Barhl1* was due to lack of proliferation because there was no difference in the percentage of phospho-histone H3 positive cells between normal and *Bmpr* mutant littermates (Table 1).

In a previous analysis of dorsal-ventral patterning in the caudal neural tube at the level of the spinal cord, we have demonstrated an increase in the number of dorsal-ventral interneuron precursors (DI3 and DI4; [40]). In Fig 2, we observed a dorsal expansion of equivalent interneuron subtypes (Lhx1/5 & Pax2 positive cells) consistent with the hypothesis that similar changes are occurring in the hindbrain.

*Ptf1a* is expressed in a domain that lies ventral to the expression domain of *Atoh1*-postive precursors that give rise to precerebellar mossy fiber nuclei, and is responsible for specifying climbing fibers derived from the ION [23]. In the *Bmpr* double knockout mutants, the *Ptf1a* domain is shifted dorsally to abut the roof plate. Despite this shift in the *Ptf1a* domain, we have not observed major changes in the size or morphology of the ION (Figs 4 and 5). Given the absence of apoptotic cells in the dorsal neural tube, the absence of change in cell proliferation and the dorsal shift in the expression of *Ptf1a*, our working hypothesis is that in the absence of BMP signaling, the most dorsal alar plate neural precursors are specified to become more ventral *Ptf1a*-positive neural precursors. *Ptf1a* and *Atoh1* mutually negatively regulate their expression [64]. We hypothesize that without *Atoh1* expression to negatively regulate *Ptf1a*, cells that would normally express *Atoh1* express *Ptf1a* instead, causing a dorsal shift of *Pft1a* in *Bmpr* double knockout mutants.

### Role of BMP signaling in regulating Wnt expression in the hindbrain

BMPs and several members of the Wnt gene family, including *Wnt1*, are expressed in the roof and/or dorsal alar plate [47], where they regulate cell development [49, 65–67]. Wnt proteins regulate cell fate decisions, cell polarity, and embryonic patterning [25, 68, 69]. More specifically, previous evidence indicates that Wnt signaling plays a critical role in the specification of cell types for dorsal interneuron in the spinal cord and that Wnt proteins are direct regulators in the determination of dI1-dI3 interneurons [70]. Absence of *Wnt1* leads to diminished development of dI1-dI3 neurons and a compensatory increase in Lim1/Pax2 double positive (presumably dI4 or dI6) neuron populations [71]. Thus, the dorsal neural tube coordinates growth and pattern formation by the production of two classes of signaling pathways, BMPs and Wnts.

Similar to its role in the spinal cord, Wnt1 plays a crucial role in the specification of the most dorsal cell types, which give rise to precerebellar progenitor cells of the caudal rhombic lip. *Wnt1* is expressed along the rhombic lip in a dorsal-ventral gradient. The precerebellar progenitor neurons originate within the *Wnt1* expressing caudal rhombic lip and are spatially and molecularly well-defined. The mossy fiber precerebellar neurons which contribute to the PN, RtTg, ECu, and LRt originate from the dorsal domain of the caudal rhombic lip specified by high expression of *Wnt1* and the expression of *Atoh1* (Fig 1E and arrowhead in Fig 6A; [15]). Climbing fiber neurons which contribute to the ION are derived from a more ventral domain of the caudal rhombic lip which expresses a low level of *Wnt1* and also expresses *Ptf1a* (Fig 5A and arrow in Fig 6A; [2]).

Our results demonstrate that the domain of *Wnt1* expression in the caudal rhombic lip is reduced in *Bmpr* double mutant animals (Fig 6A and 6B). Even though *Wnt1* expression domain overlaps *Atoh1* positive domain, we hypothesize the loss of *Wnt1* expression is not due to a secondary effect from the absence of *Atoh1* expression. *Wnt1* expression is lost in both *Atoh1* and *Ptf1a* positive cells and not just *Atoh1* positive cells (Fig 6A and 6B) even though only *Atoh1* expression is lost in *Bmpr* double knockout mutants whereas *Ptf1a* expression is not lost (Fig 5A and 5B). In *Wnt1* knockout mutants, *Atoh1* is decreased [66]. Furthermore, it has been shown that *ß-catenin*, a downstream component of Wnt signaling, binds to *Atoh1* enhancer and promotes *Atoh1* expression [72]. Also, BMP signaling was shown to up-regulate *Wnt1* expression in the chicken dorsal neural tube [70, 73]. Zechner and colleagues further demonstrated that in chick neural tube assays BMP signaling could regulate the expression of Wnt gene family members, but that Wnt expression did not have the reciprocal ability to regulate BMP expression. Results presented here demonstrate that BMP signaling is epistatic to the expression of Wnt1 gene from the roof and dorsal alar plate of the hindbrain and is necessary for the development of mossy fiber precerebellar progenitors.

Interestingly, the non-*Gdf7* positive roofplate is reduced in the *Bmpr* double knockout mutant (arrowhead in Fig 6C and 6D), similar to the reduction of non-*Gdf7* positive roofplate observed in the *Lmx1a* mutant [74]. We hypothesize that this reduction is caused by a drastic reduction in *Lmx1a* expression in the *Bmpr* mutant caudal rhombic lip (Fig 6E and 6F). The reduced *Lmx1a* expression may not contribute significantly to the lack of specification of mossy fiber progenitors seen in the *Bmpr* mutant, because *Atoh1*, *Gdf7*, and *Wnt1* are present in the caudal rhombic lip of *Lmx1a* mutants (Fig 6 of [74]). Only by ablating the *Gdf7* positive roofplate does one completely lose *Atoh1*, *Gdf7*, and *Wnt1* expression in the caudal rhombic lip (Fig 7 of [74]) and the *Gdf7* positive roofplate is present in the *Bmpr* double knockout (Fig 6D).

## Conclusion

Our results demonstrate that BMP signaling is involved in the generation of the pontine gray nuclei and other precerebellar nuclei that generate mossy fiber input to the cerebellum. Furthermore, BMP signaling, through *Wnt1* expression, specifies the *Atoh1* positive mossy fiber progenitor cells. These analyses with double knockouts of the *Bmpr1a* and *Bmpr1b* genes demonstrate the importance of BMP signaling for appropriate expression of *Wnt1* and the development of the precerebellar system in the hindbrain.

## Supporting information

S1 Fig Gross neuroanatomy of the *Bmpr1a* single mutants are unaffected beyond distortions introduced by hydrocephalus. Horizontal sections of normal (A) and *Bmpr1a* conditional knockout (B) animal brains.
(TIF)

S2 Fig Labeling of molecular markers for pontine nuclei at P0. (A, B) Zic1/2 immunostaining was observed in the pontine nuclei of normal animals (A), while the number of Zic-positive cells was reduced in the *Bmpr* double knockouts (B). (C, D) To examine the expression of *Pax6*, in situ hybridization analyses were undertaken. The number of *Pax6*-labeling cells was greater in normal animals (C, arrow) than in *Bmpr* double knockout animals (D). Scale bar: A (for A-F), 250 μm.
(TIF)
