# Supplementary figures and images for: Analyses with double knockouts of the Bmpr1a and Bmpr1b genes demonstrate that BMP signaling is involved in the formation of precerebellar mossy fiber nuclei derived from the rhombic lip
Source: PLoS One. 2019 Dec 23;14(12):e0226602. doi: 10.1371/journal.pone.0226602 (PMC6927620; doi:10.1371/journal.pone.0226602)

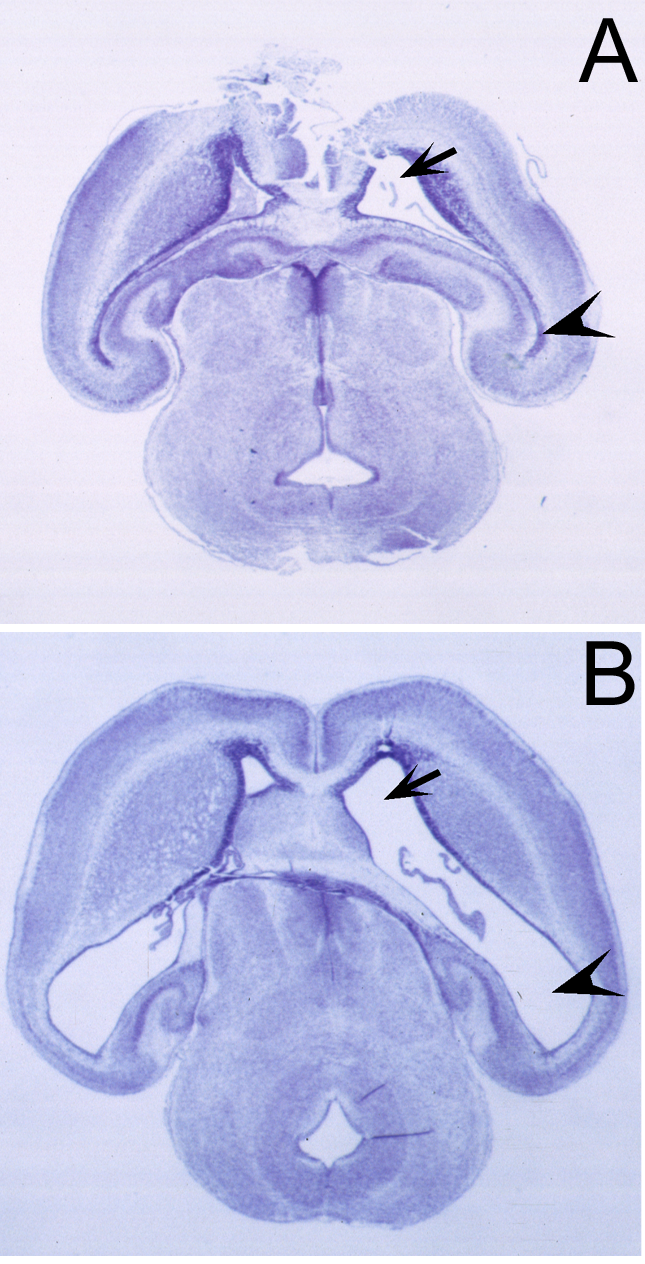

Supplement: S1 Fig — Horizontal sections of normal (A) and Bmpr1a conditional knockout (B) animal brains. (TIF) [file pone.0226602.s001.tif]

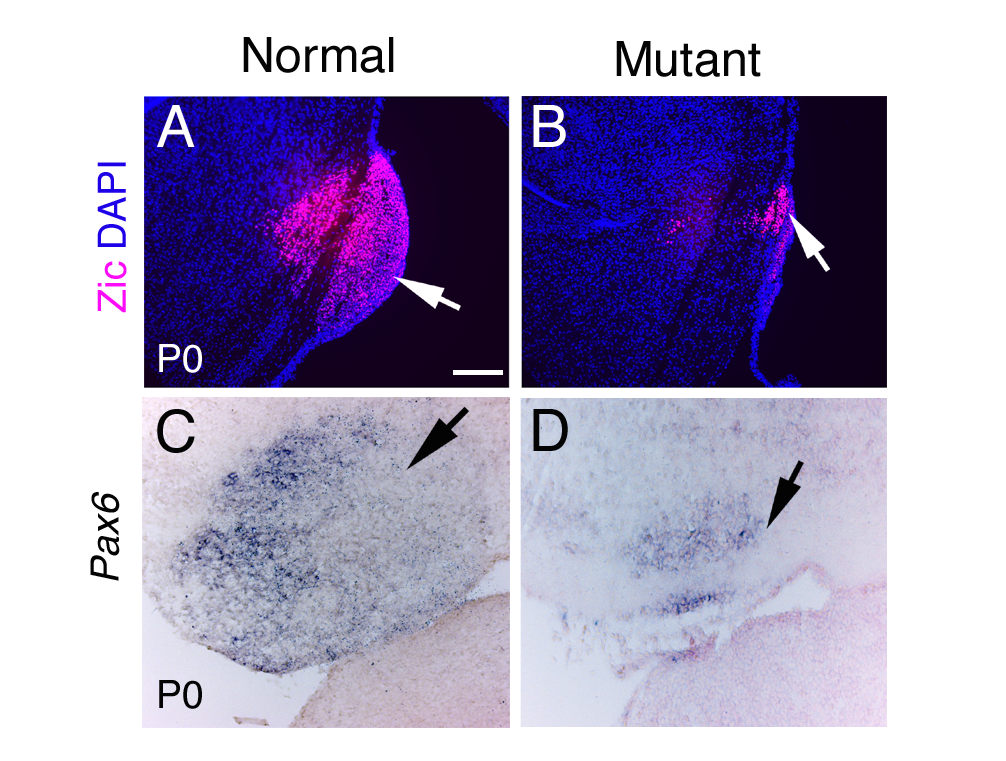

Supplement: S2 Fig — (A, B) Zic1/2 immunostaining was observed in the pontine nuclei of normal animals (A), while the number of Zic-positive cells was reduced in the Bmpr double knockouts (B). (C, D) To examine the expression of Pax6, in situ hybridization analyses were undertaken. The number of Pax6-labeling cells was greater in normal animals (C, arrow) than in Bmpr double knockout animals (D). Scale bar: A (for A-F), 250 μm. (TIF) [file pone.0226602.s002.tif]
